# Supplementary material for: Analysis of interaction dynamics and rogue wave localization in modulation instability using data-driven dominant balance
Source: Sci Rep. 2023 Jun 28;13:10462. doi: 10.1038/s41598-023-37039-7 (PMC10307870; doi:10.1038/s41598-023-37039-7)
Supplement: Supplementary file 1 — Supplementary Legend. [file 41598_2023_37039_MOESM1_ESM.docx]

Analysis of interaction dynamics and rogue wave localization in modulation instability

using data-driven dominant balance

A. Ermolaev et al.

**Legend**

To complement Figure 1 of the main manuscript, a Supplementary Video S1 (.mov file) shows an animation of the evolution of the intensity profile of the Peregrine soliton, as well as the different terms and the associated color-coded clusters in equation space.
